# Beyond Face and Voice: A Review of Alexithymia and Emotion Perception in Music, Odor, Taste, and Touch

**DOI:** 10.3389/fpsyg.2021.707599

**Published:** 2021-07-30

**Authors:** Thomas Suslow, Anette Kersting

**Affiliations:** Department of Psychosomatic Medicine and Psychotherapy, University of Leipzig Medical Center, Leipzig, Germany

**Keywords:** alexithymia, emotion perception, music, olfaction, gustation, touch

## Abstract

Alexithymia is a clinically relevant personality trait characterized by deficits in recognizing and verbalizing one's emotions. It has been shown that alexithymia is related to an impaired perception of external emotional stimuli, but previous research focused on emotion perception from faces and voices. Since sensory modalities represent rather distinct input channels it is important to know whether alexithymia also affects emotion perception in other modalities and expressive domains. The objective of our review was to summarize and systematically assess the literature on the impact of alexithymia on the perception of emotional (or hedonic) stimuli in music, odor, taste, and touch. Eleven relevant studies were identified. On the basis of the reviewed research, it can be preliminary concluded that alexithymia might be associated with deficits in the perception of primarily negative but also positive emotions in music and a reduced perception of aversive taste. The data available on olfaction and touch are inconsistent or ambiguous and do not allow to draw conclusions. Future investigations would benefit from a multimethod assessment of alexithymia and control of negative affect. Multimodal research seems necessary to advance our understanding of emotion perception deficits in alexithymia and clarify the contribution of modality-specific and supramodal processing impairments.

## Introduction

The personality construct of alexithymia refers to deficits in the cognitive processing of emotions comprising difficulties in identifying and describing feelings, an externally oriented thinking style focused on concrete details of events and constricted imaginal processes (Taylor and Bagby, 2000). Prevalence studies indicate that ~10% of the general population are affected by clinically relevant alexithymia (Mattila et al., 2006; Franz et al., 2008). Alexithymia is a major risk factor for a range of somatic and mental disorders (Leweke et al., 2012; Porcelli and Taylor, 2018). Experiences of childhood maltreatment (especially emotional neglect and emotional abuse) seem to be an important environmental factor for the development of adult alexithymia (Aust et al., 2013; Brown et al., 2018).

Importantly, alexithymia has been found to be associated with deficits in perceiving other persons' emotions, e.g., from facial expressions (Parker et al., 1993). The ability to understand accurately emotional facial expressions is of primary importance for humans to interact successfully with one another (Erickson and Schulkin, 2003). An impaired perception of facial emotional signals can result in dysfunctional interpersonal patterns, which are frequently observed in alexithymic individuals (Spitzer et al., 2005). The study of alexithymia and emotion recognition seems useful to open up new perspectives in understanding the vulnerability mechanisms leading to clinical disorders. Specifically, a better comprehension of the links between alexithymia and emotion perception could help to tailor more specific therapeutic approaches that improve social skills in alexithymic individuals.

Emotion perception can be defined as processes that comprise the identification of emotionally salient information in the environment and the generation of emotional experiences and behavior in response to this information (Phillips, 2003). Exteroceptive senses by which humans perceive emotional information in the outside world are sight, hearing, taste, smell, and touch. Recent psychological research has developed complex models of emotion recognition abilities considering modality-specific and superordinate cognitive processes (Lewis et al., 2016; Connolly et al., 2020). Evidence has been found for different levels and processing components within the functional architecture of emotion recognition (Lewis et al., 2016; Connolly et al., 2020). A supramodal emotion recognition factor can be differentiated from subordinate modality-specific factors.

From a neurophysiological perspective, sensory systems appear at first sight relatively independent of one another. They include specialized sensory organs and receptors, for example, in the ear, nose, mouth, and skin that correspond to a respective auditory, olfactory, gustatory, and somatosensory system (Moller, 2002). Sensory receptors are connected with specific areas of the brain where the sensory information is processed, the primary and secondary cortices of the different senses. Processing of signals across the different sensory modalities converges into multi-modal and then into amodal representations that enable emotion judgments. The medial prefrontal, the posterior superior temporal and the posterior cingulate cortex seem to represent perceived emotional information at an abstract, modality-independent level and to play a key role in its linguistic categorization (Peelen et al., 2010; Klasen et al., 2011). However, mixing of modalities is assumed to begin during early perceptual stages in the brainstem and thalamus, and to progress at the level of the cerebrum, where information is integrated and mapped onto existing emotion templates (Schirmer and Adolphs, 2017). Thus, higher-level representations are thought to feedback and modulate lower-level perceptual representations.

There is evidence that basic emotions (such as anger, fear, sadness, and happiness) can be more easily expressed, evoked, and perceived in the visual, auditory, and tactile modality (Keltner et al., 2019) than in the olfactory or gustatory modality (Shepherd, 2006; Croy et al., 2011). Perceptual representations of odors and tastes might carry simpler information on affective qualities. Olfactory and taste stimuli have been shown to elicit primarily the emotions of pleasure/happiness and disgust (Shepherd, 2006; Croy et al., 2011) and might be better described using a pleasantness-unpleasantness continuum than concepts of basic emotions. Against this background, it is not surprising that, for example, in studies on auditory emotion perception individuals are frequently asked to decide which emotion quality is expressed in a soundtrack whereas in studies on gustatory or olfactory emotion perception subjects should rate the intensity or hedonic valence of stimuli.

In the last decades, a considerable number of studies investigated the relationship between alexithymia and emotion face perception. Alexithymia research focused on the perception of emotions from faces as emotional facial expressions are encountered often in everyday life (Calvo et al., 2014), and their controlled presentation in experiments is rather simple especially when standardized stimulus sets are used. Although all emotional expressions extend in time faces can be identified from a single snapshot whereas the study of emotional vocalizations or touch is more complex and requires dynamic stimuli for which receivers must integrate information over time. A key finding from research on alexithymia and emotion face perception has been that alexithymic individuals have deficits in identifying others' facial emotions that are neither emotion-specific nor valence-specific (see Grynberg et al., 2012, for a review). There is evidence that these recognition deficits could be more pronounced and therefore easier to detect under suboptimal presentation conditions, e.g., when faces are shown in degraded quality (Kätsyri et al., 2008) or with temporal constraints (Ihme et al., 2014). Previous research on alexithymia and emotion perception has been clearly dominated by facial emotions, with other sensory modalities explored less frequently. The second most frequently studied modality in alexithymia research on perception is speech or prosody. In humans, non-verbal vocalizations of basic emotions are universally recognized (Sauter et al., 2010). Alexithymia was found to be associated with a reduced capacity to recognize emotions from vocal expressions in healthy individuals and patients with mental disorders (Goerlich et al., 2011; Heaton et al., 2012; Bayot et al., 2014 but see Swart et al., 2009, for null results).

Previous psychological research investigating emotion perception across different domains and modalities (face, body, and voice) and levels of processing (domain- or modality-specific and supramodal) revealed primarily associations between alexithymia and supramodal emotion recognition (Lewis et al., 2016; Connolly et al., 2020). However, there was also evidence supporting a relationship of alexithymia with modality-specific (i.e., face-specific) emotion recognition (Lewis et al., 2016). These results indicate that, on the one hand, alexithymia may be associated with deficits in the multi-sensory integration and conceptual representation of emotional information (which also includes the sorting into emotion categories defined by words in a particular language). On the other hand, it suggests that alexithymic individuals could also have problems in identifying emotional information within a specific modality or domain of emotion recognition. A rather consistent finding from functional neuroimaging research is a reduced neural activity in several relevant brain areas during emotion processing in alexithymia (Goerlich and Aleman, 2018). Alexithymic individuals manifest a decreased responsivity in the amygdala, insula, orbitofrontal and medial prefrontal areas. The medial prefrontal cortex is assumed to play a key role in the categorization of emotional information and affective decision-making (Harris et al., 2007; Etkin et al., 2011). Across different sensory modalities (e.g., taste, olfaction, and touch), amygdala and orbitofrontal cortex have been shown to be crucially involved in determining the basic reward and affective value of environmental stimuli (Rolls and Grabenhorst, 2008). Low neural reactivity to emotional stimuli in these brain areas may result in deficits in the ability to represent and identify their emotional quality. Moreover, alexithymia has been found to be associated with reduced gray matter volumes in the amygdala, insula, and the orbitofrontal cortex (see Xu et al., 2018, for a recent meta-analysis). These cerebral volume reductions could contribute to alexithymic individuals' deficiencies in appropriately identifying emotions. Interestingly, findings from a recent review on electrophysiological research (Goerlich, 2018) suggest that emotion processing impairments in alexithymia appear not restricted to late, conscious processing stages, but begin already in a time window of earlier than 100 ms after stimulus presentation. Goerlich (2018) concludes that the emotion processing difficulties in alexithymia seem to be deep-seated in automatic processes of emotion perception and may affect specific sensory domains as well as the multi-sensory integration of emotional information. The early processing deficits are assumed to contribute to the deficits in late stages of emotion processing (Donges and Suslow, 2017).

The objective of our review was to summarize and systematically assess the literature on the effects of alexithymia on the perception of emotional (or hedonic) stimuli in the modalities or expressive domains music, olfaction, touch, and gustation. Such an overview can help to clarify whether alexithymia adversely affects various modalities of emotion perception and whether this impact occurs similarly across valence and emotion quality conditions. To this aim, the Web of Science and PsychINFO databases were searched up to and including December 2020. Emotional stimuli are defined as environmental stimuli that are capable of eliciting emotional reactions or that inform about the emotional state of other persons. Most emotion theories concur that emotional stimuli are a special type of stimulus possessing high relevance for the wellbeing of the observer (Brosch et al., 2010).

## Literature Search: Search Strategy, Selection Criteria and Procedure

Web of Science and PsychINFO databases were searched until December 2020 using the keywords (“alexithymi^*^”) AND (“perception” OR “processing”) AND (“emotion^*^”) AND (“music^*^”, OR “odor”, OR “odour”, OR “smell,” OR “olfactory”, OR “taste”, OR “gustatory”, OR “touch”, OR “tactile”, OR “haptic”). Only *original* research articles were included in our review that tested emotion perception abilities experimentally. Exclusion criteria were review articles, opinions, commentaries, study protocols, and letters to the editor. Studies published in languages different from English were excluded. Upon search completion, titles and abstracts of the identified articles were assessed for their suitability to be included in this review. Only those studies were considered in which alexithymia was assessed on the basis of standardized psychometric instruments. After analyzing titles and abstracts, the full text of the articles considered suitable were retrieved for further examination of the investigations to determine their final inclusion in our review. The reference lists of the selected articles were also examined for suitable publications that might have been overlooked in the previous search.

In the large majority of studies, the TAS-20 (or TAS-26) has been used as the only or main instrument for the assessment of alexithymia (see Table 1, last column). In the following study descriptions, we will mention the administered alexithymia measure in case an instrument other than the TAS was applied.

**Table 1:** Summarizing overview of the studies on alexithymia and emotion perception included in the present review.

**Sensory modality/domain**	**References**	**Emotion qualities of stimuli**	**Population**	**Sample size**	**Alexithymia** ** measure**
Audition/Music	Allen et al., 2013	s, h, sc (30 s music clips)	Autism	23	BVAQ
			Healthy	24	
	Goerlich et al., 2011	s, h (0.6 s recordings of classical music)	Healthy	32	TAS-20
	Larwood et al., 2021	a, f, s, h, t (15 s soundtracks)	Healthy	162	TAS-20
	Punkanen et al., 2011	a, f, s, h, t (15 s soundtracks)	Depressed	79	TAS-20
	Taruffi et al., 2017	a, f, s, h, t (15 s soundtracks)	Healthy	120	TAS-20
Olfaction	Cecchetto et al., 2017	Unpleasant (butyric acid)	Healthy	62	TAS-20, BVAQ
	Lombion et al., 2010	Unpleasant (Tetrahydrothiophene)	Depressed, anorectic	24	TAS-20
		Pleasant (L-carvone)	Healthy	9	
	Özsoy-Ünübol et al., 2020	Pleasant (phenylethyl alcohol)	Fibromyalgia	30	TAS-20
			Healthy	20	
Gustation	Özsoy-Ünübol et al., 2020	Sweet, bitter, sour, salty	Fibromyalgia	30	TAS-20
			Healthy	20	
	Robino et al., 2016	Bitter (PROP, 6-*n*-Propylthiouracil)	Healthy	649	TAS-20
Touch	Borhani et al., 2017	Stroking of the forearm	Healthy	40	TAS-20, DCPR
	Iosifyan and Korolkova, 2019	a, f, h, d, s, su; touching textures	Healthy	108	TAS-26

*Emotion qualities: a, angry; d, disgusting; f, fearful; h, happy; s, sad; s, scary; su, surprising; t, tender; TAS-20, 20-Item Toronto Alexithymia Scale (Bagby et al., 1994); BVAQ, Bermond-Vorst Alexithymia Questionnaire (Vorst and Bermond, 2001); DCPR, Diagnostic Criteria for Psychosomatic Research (Fava et al., 1995); TAS-26, Toronto Alexithymia Scale 26-Item version (Taylor et al., 1985)*.

## Alexithymia and Emotion Perception in Music

Music is frequently defined as sound events in time. It can express basic human emotions (Mohn et al., 2011). Already young children can recognize emotions in music (Nawrot, 2003). The universal capacity to identify emotional expressions in music was shown in cross-cultural studies (Fritz et al., 2009).

Goerlich et al. (2011) applied a cross-modal priming task to investigate automatic processing of emotional music stimuli as a function of alexithymia in a sample of healthy individuals. Here, short recordings of sad and happy classical music (with a duration of 600 ms) were administered as prime and target stimuli (along with positive and negative words). According to the behavioral results, there was no effect of alexithymia on affective priming for music targets or on words primed by emotional music. Thus, no relationship was found between alexithymia and automatic affective categorization of music.

Allen et al. (2013) used music clips that represented the emotions happy, sad, and scary. The musical stimuli had been developed and validated by Quintin et al. (2011). The music clips were presented to individuals with autism and healthy controls under two conditions. First, on the basis of a list of emotion words they had to describe the way the music makes them feel (with the comment that this may not be the feeling one sees expressed by the music). Second, participants were asked to decide which group of words had been used previously by a group of people to describe the way they felt about the music presented. The Bermond-Vorst Alexithymia Questionnaire (BVAQ; Vorst and Bermond, 2001) was administered to assess alexithymia. The autism group showed a reduced level of verbal emotional responsiveness to the music items compared to the control group. However, when type II alexithymia (as measured by the relevant BVAQ factors) was controlled the between-group difference became non-significant. Type II alexithymia is defined by a normal range of emotions experienced and an impaired cognitive emotion processing (i.e., identification, analysis, and verbalization of emotions; Bermond et al., 2007). Negative correlations between type II alexithymia and verbal emotional responsiveness were revealed. No group difference was observed for the judgements which emotion words had been used by other people to describe the way they felt about the music presented. In this latter case, Allen et al. did not analyze the relationship between alexithymia and the supposed emotional response of others.

In the study of Punkanen et al. (2011), depressed patients heard soundtrack clips expressing five emotions (anger, fear, sadness, happiness, or tenderness). The patients had to evaluate how much the music represents emotions. High alexithymia in patients led to a reduced perception of anger, fear, and tenderness in the soundtracks. It was concluded that alexithymia seems to flatten the emotional perception of music in depression.

Taruffi et al. (2017) also used soundtrack clips expressing anger, fear, sadness, happiness, or tenderness as music stimuli. After listening to each musical excerpt healthy subjects had to select one out of five emotions that they thought the music aimed to convey. The alexithymia component externally oriented thinking was found to be negatively linked to the total score of musical emotion recognition. On the level of single emotions, externally oriented thinking was associated with less recognition of sadness and tenderness in music. The authors assumed that individuals with an externally oriented thinking style could avoid processing emotional information.

In the study of Larwood et al. (2021), healthy individuals were instructed to judge the affective valence and arousal of soundtrack clips expressing anger, fear, sadness, happiness, or tenderness. Higher alexithymia predicted more neutral judgements of valence and arousal when the target emotion of the song was negative. Alexithymia seems to be related to less intense perceptions of sad, angry, and fearful music. It is suggested that the dampening of affective judgements when faced with negatively valenced music might be a product of a diversion of attention away from unpleasant emotional cues.

Taken together, data from four out of five studies suggest that alexithymia is related to abnormalities in the perception of emotions in music (Punkanen et al., 2011; Allen et al., 2013; Taruffi et al., 2017; Larwood et al., 2021). The perception of negative but also positive emotions seems to be impaired in alexithymia. However, the observed deficits concern primarily the recognition and under-attribution of sadness and threat-related emotions to emotional music. The findings on alexithymia and emotion perception in music appear rather consistent across studies considering that Goerlich et al. (2011) presented recordings of music for only 0.6 s in an affective priming paradigm and examined automatic (but not controlled) processes of emotion recognition. Of note, three studies (Punkanen et al., 2011; Taruffi et al., 2017; Larwood et al., 2021) applied stimulus material from the film soundtrack list provided by Eerola and Vuoskoski (2011) and used a rather long stimulus duration (i.e., 15 s). The results of Allen et al. (2013) suggest that individuals with impaired cognitive emotion processing manifest a low level of verbal emotional responsiveness to music.

## Alexithymia and Olfactory Emotion Perception

Odor perception is regulated by evolutionary old brain areas that are closely linked to the limbic system. Odors can elicit strong emotional responses and are known to be an integral part of emotional memories (Herz, 2016).

In the study of Lombion et al. (2010), odor perception was investigated in alexithymic and non-alexithymic patients suffering from major depression or anorexia nervosa and a group of healthy controls. Tetrahydrothiophene (an unpleasant odor) and L-carvone (a sweetish minty smell) were used as odors to examine olfactory sensitivity and perception of intensity and hedonic valence. No group differences were observed in the ability to detect odors in the sensitivity test. Alexithymic patients were found to overrate intensity and pleasantness of odors compared with non-alexithymic patients and control subjects. This over-evaluation of intensity and hedonicity could be interpreted in the framework of a deficit of cortical inhibition shown by alexithymics.

Cecchetto et al. (2017) examined the effect of alexithymia on olfactory emotion perception in a sample of healthy individuals. All participants were tested for odor threshold perception and asked to evaluate intensity and hedonic valence of unpleasant odor (butyric acid), neutral odor (gardenia essential oil) and clean air. Study participants were divided into three groups on the basis of their BVAQ scores as proposed by Deborde et al. (2008): low, medium, and high alexithymic subjects. No group differences were observed for odor detection and identification performance and pleasantness or intensity ratings of odors. An additional analysis of alexithymia components revealed a distinct pattern of olfactory abnormalities related to some components. Difficulties communicating emotions were related to faster pleasantness decisions in the odor-rating task. Moreover, difficulties explaining one's emotions were associated with a lower odor threshold. Evaluating different alexithymia components could be important in future research on alexithymia and olfactory emotion perception since they may affect odor processing in different ways.

Özsoy-Ünübol et al. (2020) evaluated smell functions in patients with fibromyalgia syndrome and healthy individuals. In their study, the Sniffin' sticks test (Hummel et al., 2007) was performed with phenylethyl alcohol [a pleasant (floral) odor]. In the total sample, alexithymia correlated negatively with the overall olfactory function test score. Thus, high alexithymia went along with a reduced ability in odor identification and discrimination.

In sum, there is some evidence that alexithymia could be linked to a reduced identification of pleasant odors (Özsoy-Ünübol et al., 2020; but see Lombion et al., 2010, for null results concerning odor detection). Cecchetto et al. (2017) even reported that difficulties in analyzing emotional reactions were associated with higher odor sensitivity. Thus, the available data do not provide a consistent picture of the effects of alexithymia on odor detection and identification. Abnormalities were observed in the perception of pleasantness in odors: alexithymic characteristics might be linked to an overrating of pleasantness of odors (Lombion et al., 2010) and faster pleasantness decisions (Cecchetto et al., 2017). Several factors may have contributed to the considerable heterogeneity of findings between studies. First, the studies differed in their methodology of olfactory testing but also stimulus material. Different odorant substances were administered. Some authors applied only unpleasant-smelling or pleasant-smelling odors while others administered both types of odors. Moreover, the study samples differed in mental health status (participants were healthy or suffered from major depression, anorexia nervosa, or fibromyalgia). A further possible explanation for the inconsistencies of results between studies is the use of different measures of alexithymia. Unlike the other authors, Cecchetto et al. (2017) based their analyses on the BVAQ which assesses two dimensions of alexithymia (emotionalizing and fantasizing) that are not measured by the TAS-20. Finally, it has to be noted that small sample sizes in the studies of Lombion et al. (24 depressed, anorectic and nine healthy subjects) and Özsoy-Ünübol et al. (30 fibromyalgia patients and 20 healthy subjects) may also account for the mixed results.

## Alexithymia and Gustatory Emotion Perception

Five main taste qualities are known to be perceived by humans: sweet, bitter, sour, salty, and umami (or savory) (Breslin and Huang, 2006). Both mouth and nose are involved in tasting. Bitter and sour tastes are typically perceived as unpleasant; they signal the presence of potentially harmful chemicals and alert the organism to the presence of toxic or spoiled foods (Kinnamon, 2012). Sweet taste normally reveals the presence of sugars and elicit in general positive hedonic responses (Jayasinghe et al., 2017).

Robino et al. (2016) investigated taste perception as a function of alexithymia in a large sample of healthy persons. The authors assessed the ability to perceive the bitter taste of PROP (6-*n*-Propylthiouracil). Subjects had to rate the taste intensity. Alexithymia was found to be linked to a reduced bitter taste sensitivity. The data of Robino et al. (2016) indicate that especially the alexithymia components difficulties in identifying feelings and externally oriented thinking are accompanied by a diminished perception of aversive taste. It is argued that alexithymia could be related to avoidance behavior leading to a decreased exposure to negative perceptions.

Özsoy-Ünübol et al. (2020) examined in their study also taste functions. Four concentrations of different taste strips (sweet, bitter, sour, and salty) were used for gustatory function analysis. In the total sample composed of patients with fibromyalgia and healthy individuals, alexithymia was negatively related to the overall taste function test score. That is, alexithymia seems to be connected with a diminished ability in taste identification and discrimination.

Taken together, the results from both studies yield a rather consistent picture of the effects of alexithymia on gustatory functioning. Alexithymia appears to have an adverse impact on taste perception and may in particular diminish perceptual sensitivity for aversive, bitter taste.

## Alexithymia and Tactile Emotion Perception

Perception of touch requires direct physical contact. Tactile stimulation usually occurs as a combination of an active movement (reaching out to touch a surface) and a sensation (actually feeling the surface against the skin). Touching and being touched have central roles for social development throughout the lifespan (Cascio et al., 2019). Distinct emotional states can be communicated via touch (Hertenstein et al., 2006).

Iosifyan and Korolkova (2019) examined the effect of alexithymia on emotion perception during touching of different textures. Healthy participants touched 21 tactile surfaces (e.g., sandpaper, velvet, or toy slime) and evaluated how much the textures were associated with six basic emotions. Alexithymia correlated with more intense experiences of disgust, anger, and sadness during touch of textures. These findings seem to be in line with the hyperarousal theory of alexithymia that advocates that alexithymic individuals display exaggerated physiological responses to emotional stimuli.

Borhani et al. (2017) studied whether alexithymia has an impact on the perception of affective touch. In their experiment, stroking stimuli were delivered to the forearm and participants had to rate pleasantness and softness of the tactile stimulation. No difference in the experience of pleasant affective touch between high and low alexithymic individuals was revealed. It is a strength of Borhani et al.'s study that the structured interview for the Diagnostic Criteria for Psychosomatic Research (DCPR, Fava et al., 1995) was used to confirm the presence (or absence) of alexithymia as defined by the TAS-20.

All in all, there is some evidence of an effect of alexithymia on tactile emotion perception. Alexithymia seems to be associated with more intense perceptions of negative emotions during active touch. It should be noted that the two studies on alexithymia and tactile emotion perception differ substantially in the processes examined and their evaluation procedures. This might explain the differences between findings. Iosifyan and Korolkova (2019) analyzed the touching of textures. In this case, perception is achieved through the active exploration of surfaces by a moving subject (haptic perception). Borhani et al. (2017) investigated instead the affective impact of being touched. In the latter case, subjects were static and passive during tactile perception. Iosifyan and Korolkova (2019) evaluated how much textures were associated with six basic emotions whereas Borhani et al. (2017) collected evaluative data using a dimensional pleasantness rating which requires simpler judgments.

## Conclusions and Directions for Future Research

We identified eleven relevant studies on alexithymia and emotion perception from the literature that addressed the olfactory, gustatory, tactile, or musical modality or expressive domain. The small number of studies conducted indicates that, at present, research on these topics is still in its initial stage. This could be due to the fact that this kind of perception research is technically more demanding and more expensive than studies based on established sets of standardized facial or vocal emotional expressions. However, it is important to find out whether alexithymia has a similar effect across different modalities of emotion perception and whether this impact occurs equally across valence and emotion quality conditions. To date, alexithymia research on emotion perception has emphasized the face and, in second place, the voice but there is neuroscientific evidence that each sensory modality represents a rather distinct input channel engaging neural networks with different processing specializations (Schirmer and Adolphs, 2017). Thus, each sensory modality in emotion perception should be studied in its own right.

According to the findings presented here there are rather clear indications that alexithymia could be associated with deficits in the perception of primarily negative but also positive emotions in music (Punkanen et al., 2011; Taruffi et al., 2017; Larwood et al., 2021). The observed perceptual impairments concern the recognition and under-attribution of negative emotions to music. These findings on emotion perception in music are consistent with data from research on auditory emotion perception in speech and prosody. Previous studies examining healthy individuals and patients with mental disorders have revealed that alexithymia is related to a reduced capacity to recognize emotions from vocal expressions (Goerlich et al., 2011; Heaton et al., 2012; Bayot et al., 2014). Similarities of emotion expression in speech and music have been noticed early. It is assumed that both may have their origin in nonlinguistic affective vocalizations (Scherer, 1995). Interestingly, decoding accuracy for basic emotions is similar for vocal expression and music performance (Juslin and Laukka, 2003). Neuroimaging studies on music and emotion have shown that music modulates activity in brain areas that are involved in emotion perception such as the amygdala, nucleus accumbens, insula, cingulate cortex, and orbitofrontal cortex (Koelsch, 2014). As mentioned in the introduction, structural alterations as well as functional abnormalities during (primarily visual) emotion processing have been observed for the amygdala, insula, and orbitofrontal cortex in alexithymia (Goerlich and Aleman, 2018; Xu et al., 2018). It is possible that dysfunctions in these relevant cerebral areas have led to the difficulties in the identification of basic emotions in music associated with alexithymia. However, these assumptions are of course speculative since the reviewed behavioral studies did not investigate the neural substrates of music perception. It remains unclear at which level of neural processing the functional deficits may be located. Processing of sensory signals may be already affected by functional impairments in specialized primary and secondary cortices. Early processing deficits could contribute to deficits in late stages of emotion processing. Processing deficits could occur primarily in higher-level structures such as the medial prefrontal, the posterior superior temporal, and the posterior cingulate cortex. However, the localization of functional deficits in specific brain sites seems to be difficult as higher-level areas are assumed to feedback and modulate activity in lower-level structures during emotion perception (Schirmer and Adolphs, 2017). Against this background, it will be important in future neuroimaging research on alexithymia not only to assess the responsivity of different brain structures to emotional information but also the degree of connectivity between relevant structures during emotion perception as a function of alexithymia. However, for a comprehensive understanding of the mechanisms underlying dysfunctional emotion perception in alexithymia it appears necessary to take also into consideration psychological defensive factors such as experiential avoidance of negative emotions (Constantinou et al., 2014). The avoidance of processing and experiencing negative emotion in alexithymia could be due to poor emotion regulation abilities. In addition, it may also inhibit further development of regulation skills in alexithymic individuals (Panayiotou et al., 2015).

A rather consistent picture emerges regarding the effects of alexithymia on gustatory functioning. Alexithymia may reduce perceptual sensitivity for tastes, especially aversive taste (Robino et al., 2016; Özsoy-Ünübol et al., 2020). These data are, in general, in line with those from the reviewed studies on music perception indicating that alexithymia goes along with a reduced capacity to perceive emotional information. As mentioned earlier, perceptual representations of tastes appear to carry rather simple information on affective qualities (Shepherd, 2006). Against this background, it is interesting to note that alexithymia was found to have an effect on emotion perception (in music) not only when quite complex tasks of emotion recognition (multiple-choice or multiple-rating procedures) were administered but also when rather simple perception tasks were used in which only taste intensity (but not hedonic or emotional quality) had to be rated and hedonic valence was just an implicit characteristic of the stimulus (Robino et al., 2016). The latter finding suggests that rather basic processes of gustatory emotion perception could be impaired in alexithymic individuals.

The findings of the three reviewed olfactory studies do not give a coherent picture of the effects of alexithymia on odor detection and identification. Only the data of Özsoy-Ünübol et al. (2020) are consistent with the above-mentioned results from research on emotion perception in music and tastes indicating that alexithymia is related to a reduced ability in identification and discrimination of affective odors. Possible explanations for the inconsistencies of results between the olfactory studies could be differences in odorant substances, mental health status of participants, alexithymia tests administered, and small sample sizes in two studies (Lombion et al., 2010; Özsoy-Ünübol et al., 2020).

Finally, the available data from research on tactile perception suggest that alexithymia could go along with more intense experiences of negative emotions during active touch (Iosifyan and Korolkova, 2019) but there is no evidence that alexithymia has an impact on passive perception of affective touch (Borhani et al., 2017). The discrepant results between the two studies on tactile emotion perception could be explained by differences in the perceptual processes investigated (active touching vs. being touched). Possibly, alexithymia is not associated with alterations in the perception of affective touch. However, before firm conclusions can be drawn studies have to be conducted which require identification of a wide range of emotions communicated via touch (see Hertenstein et al., 2006). The observation by Iosifyan and Korolkova (2019) that alexithymia goes along with increased negative emotional experiences during touch is, at least in part, compatible with the finding of Cecchetto et al. (2017) who found an association of the alexithymia facet difficulties explaining one's emotions with a lower affective odor threshold. These data suggest an increased sensory sensitivity to emotional information in alexithymia. According to Jakobson and Rigby (2021) individuals with difficulties in identifying and describing their feelings report being easily overwhelmed or made uncomfortable by sensory stimulation. Heightened sensory sensitivity could interfere with the recognition of one's emotions by disrupting embodiment or making it difficult to link the emotional response that they consciously experience with the environmental stimulus that triggered the response.

However, since in the study of Iosifyan and Korolkova (2019) depression was not controlled no firm conclusions can be drawn from these data. It has been shown that difficulties identifying and describing feelings can be strongly linked to depressive symptoms (Li et al., 2015; Suslow and Donges, 2017). Depression is known to be associated with negative biases in perception and interpretation (Kircanski et al., 2012). From all reviewed studies only Cecchetto et al. (2017) and Larwood et al. (2021) controlled depression as a confounding factor. This is an important methodological point that has to be taken into consideration in future studies on alexithymia and emotion perception.

A weak point of many studies is the exclusive use of self-report to assess alexithymia. Doubts have been expressed about the validity of self-report tests assessing alexithymia, as such instruments seem to depend on the ability to describe one's emotional state accurately (Lane et al., 1997). In all but one of the reviewed studies, the Toronto Alexithymia Scale was administered (see Table 1). In one study (Cecchetto et al., 2017), an additional self-report scale, the BVAQ (Vorst and Bermond, 2001), was applied and in one study the BVAQ was the only alexithymia measure administered (Allen et al., 2013). Only one study (Borhani et al., 2017) administered the structured interview for the Diagnostic Criteria for Psychosomatic Research (DCPR; Fava et al., 1995) to assess presence of alexithymia. Future research in the field should follow a multi-method approach and include also standardized interviews or reports of relatives or friends. But it must also be noted that in previous studies on alexithymia and emotion perception in which interview- or observer-rated measures were applied in addition to self-report tests self-reported alexithymia was found to be a better predictor of emotion processing than interview measures or observer ratings (Ihme et al., 2014; Brandt et al., 2015; Rosenberg et al., 2020). In our view, there is a need to develop more differentiated alexithymia measures which assess for each of the basic emotions separately whether or to what extent difficulties in identifying or describing an emotion exist. Experiences of specific patterns of restrictive emotionality during socialization might lead to selective deficits in identifying and expressing emotions (Levant et al., 2009).

Recently, emotion perception studies based on faces, body postures, and voices revealed that individual differences in emotion recognition abilities operate at modality-specific and supramodal levels (Lewis et al., 2016; Connolly et al., 2020). Alexithymia was found to be associated primarily with supramodal but also with modality-specific emotion recognition abilities (Lewis et al., 2016; Connolly et al., 2020). To enhance our understanding of the neurocognitive deficits and substrates of alexithymia it is necessary to conduct studies in which several domains or modalities of emotion perception are investigated at the same time (e.g., tactile, auditory, and olfactory stimuli). The field has to move from a unimodal to a multi- and supramodal perspective. In this way, it will also gain ecological validity since in real life situations emotion communication often occurs simultaneously via several expressive domains.

On the basis of the reviewed research, it can be preliminary concluded that alexithymia might be associated with deficits in the perception of primarily negative but also positive emotions in music and a reduced perception of aversive taste. The results of the existing studies on emotion perception in odors and touch are rather inconsistent which is probably due to the considerable differences in the applied methods and investigated processes. The small number of currently available studies on gustatory, olfactory, and tactile emotion perception in alexithymia shows that research in the field is just beginning. Future investigation on olfactory and gustatory perception should present negative and positive hedonic smells and tastes and analyze the performance and experience data separately for each valence condition.

## Author Contributions

TS and AK conceptualized the outline for this review. TS wrote the manuscript with revisions and contributions from AK. All authors contributed to the article and approved the submitted version.

## Conflict of Interest

The authors declare that the research was conducted in the absence of any commercial or financial relationships that could be construed as a potential conflict of interest.

## Publisher's Note

All claims expressed in this article are solely those of the authors and do not necessarily represent those of their affiliated organizations, or those of the publisher, the editors and the reviewers. Any product that may be evaluated in this article, or claim that may be made by its manufacturer, is not guaranteed or endorsed by the publisher.

## Abbreviations

BVAQ: Bermond-Vorst Alexithymia Questionnaire
DCPR: Diagnostic Criteria for Psychosomatic Research
TAS-20: 20-Item Toronto Alexithymia Scale
TAS-26: 26-Item version of the Toronto Alexithymia Scale.
